# Phenotypic Spectrum of CASPR2 and LGI1 Antibodies Associated Neurological Disorders in Children

**DOI:** 10.3389/fped.2022.815976

**Published:** 2022-04-07

**Authors:** Yan Jiang, Chengbing Tan, Tingsong Li, Xiaojie Song, Jiannan Ma, Zhengxiong Yao, Siqi Hong, Xiujuan Li, Li Jiang, Yuanyuan Luo

**Affiliations:** Department of Neurology, Children’s Hospital of Chongqing Medical University, National Clinical Research Center for Child Health and Disorders, Ministry of Education Key Laboratory of Child Development and Disorders, Chongqing Key Laboratory of Translational Medical Research in Cognitive Development and Learning and Memory Disorders, Chongqing, China

**Keywords:** neurological disorder, leucine-rich glioma-inactivated protein 1, contactin-associated protein-like 2, double-positive, children

## Abstract

**Objectives:**

The clinical data of patients with double-positive for leucine-rich glioma-inactivated protein 1 (LGI1) and contactin-associated protein-like 2 (CASPR2) antibodies is limited, particularly for children. This study aimed to investigate and summarize the clinical features and long-term prognosis of children’s LGI1 and CASPR2 antibodies related to neurological disorders.

**Methods:**

We collected the clinical data and prognosis of patients with dual positive antibodies of CASPR2 and LGI1, hospitalized in the Department of Neurology, Children’s Hospital of Chongqing Medical University. Furthermore, we summarized the clinical phenotypes of this disorder in children by reviewing the published literature.

**Results:**

Two patients presenting with variable neurological symptoms including pain, hypertension, profuse sweating, irritability, and dyssomnia from Children’s Hospital of Chongqing Medical University were enrolled in this study. Together with the two patients, we identified 17 children with dual CASPR2 and LGI1 antibodies, including 12 males and 5 females. At the onset, the median age was 4.1 years (range 1–16, interquartile range 2.5–13.5), with 9 children younger than 5 years and 6 adolescents. Of the 17 patients, 11 were diagnosed with Morvan syndrome, 4 with acquired neuromyotonia, 1 with Guillain-Barré syndrome, and 1 with Guillain-Barré syndrome combined with Morvan syndrome. Dysautonomia (14/17, 82.3%), pain (13/17, 76.4%), sleep disorders (13/17, 76.4%), encephalopathy (12/17, 70.5%), and weight loss (10/17, 58.8%) were the most frequently described symptoms overall. No tumors were identified. Of the 17 patients, 13 received immunotherapy comprising IVIG combination of IVMP during the acute symptomatic phase followed by oral prednisolone to maintain remission (*n* = 7), the combination of IVIG, IVMP, oral prednisolone and methotrexate (*n* = 1), the combination of IVIG, IVMP, and mycophenolate mofetil (*n* = 1), the combination of IVIG, IVMP, oral prednisolone, and rituximab (*n* = 1), IVIG only (*n* = 2), IVMP only (*n* = 1). Median modified Rankin Scale (mRS) scores in the acute phase were 3 (range 1–4) and improved gradually. Over the follow-up (median 8.6 months, range 1–36 months), 52.9% (9/17) of the patients recovered completely; one patient relapsed and showed immunotherapy-dependent.

**Conclusion:**

LGI1 and CASPR2 double-positive antibodies associated with the neurological diseases can occur in children of all ages and involve multiple nervous systems. Morvan syndrome is the most common phenotype of this disorder. The long-term outcomes are mostly favorable upon immunotherapy.

## Introduction

Leucine-rich glioma-inactivated protein 1 (LGI1) and contactin-associated protein-like 2 (CASPR2), parts of the voltage-gated potassium channel complex (VGKC-complex), are widely expressed in the central nervous system (CNS) and peripheral nervous system (PNS). LGI1 and CASPR2 antibodies have been identified in various neurological syndromes (1, 2). Anti-LGI1 antibodies are associated with limbic encephalitis, manifested with cognitive impairment, psychiatric disorders, faciobrachial dystonic seizures, and hyponatremia (3). While anti-CASPR2 antibodies are associated with different clinical spectrums, including encephalitis, acquired neuromyotonia, and Morvan syndrome (4). The two antibodies predominantly affect elderly males. In recent years, a small number of children cases of LGI1 or CASPR2 antibody-associated diseases and adults with LGI1-CASPR2 double-positive have also been reported (2, 5–7). However, the phenotypic spectrum of double-antibodies associated with neurological disorders in children has not been completely defined yet. Herein, we present two children of Morvan syndrome with LGI1-CASPR2 double-positive and perform a systematic literature review of neurological disorders in children with double antibodies positivity.

## Materials and Methods

This study is a retrospective chart review at Children’s Hospital of Chongqing Medical University. Two patients with dual positive antibodies of LGI1 and CASPR2, hospitalized in the Department of Neurology, Children’s Hospital of Chongqing Medical University from 1 January 2020 to 31 August 2021, were collected in this study. We summarized the clinical phenotypes of pediatric patients with dual positive antibodies of LGI1 and CASPR2 from the targeted literature. The literature search was carried out in PubMed,^1^ and the search deadline was 1 October 2021, with the keys words: “(LGI1 or leucine-rich glioma-inactivated protein 1)” and “(CASPR2 or contactin-associated protein 2),” “Morvan syndrome,” “Neuromyotonia.” Articles were manually searched to select pediatric patients (0–18 years of age) with dual positive antibodies to LGI1 and CASPR2 and to extract relevant information focusing on clinical data, follow-up time, and prognosis. Relapse was defined as recurrence after complete or partial recovery, with sustained improvement for at least 2 months (8). Acquired neuromyotonia is defined as a form of peripheral nerve hyperexcitability, characterized by involuntary myokymia, cramps, hypertrophy, weakness, wasting, and excessive sweating (9). Morvan syndrome is defined as an autoimmune disorder involving the peripheral, autonomic, and CNS, mainly manifesting as peripheral nerve hyperexcitability, dysautonomia, encephalopathy, and sleep disturbance (10). Guillain-Barré syndrome is defined as an acute, monophasic, peripheral nerve demyelinating disease, often presenting with progressive limb weakness with albuminocytologic dissociation of cerebrospinal fluid (CSF) (11). Modified Rankin Scale (mRS) score was used to evaluate treatment responses at diagnosis and following therapies. The Ethics Committee of the Children’s Hospital of Chongqing Medical University approved this study. All the patients’ parents or legal guardians provided informed consent to use their medical records.

## Results

### Case Presentation

#### Case 1

A previously healthy 4-year-1-month-old male presented with generalized pain, decreased appetite, fatigue, and excessive sweating, followed by irritability and weight loss, for 6 weeks before admission. During hospitalization, he developed sleep disorders, manifested by easy awakening at night. Physical examination at admission revealed normal power in all limbs, tachycardia, and arterial hypertension, with blood pressure raising to 180/115 mmHg. The mRS score at admission was 3.

**FIGURE 1:**
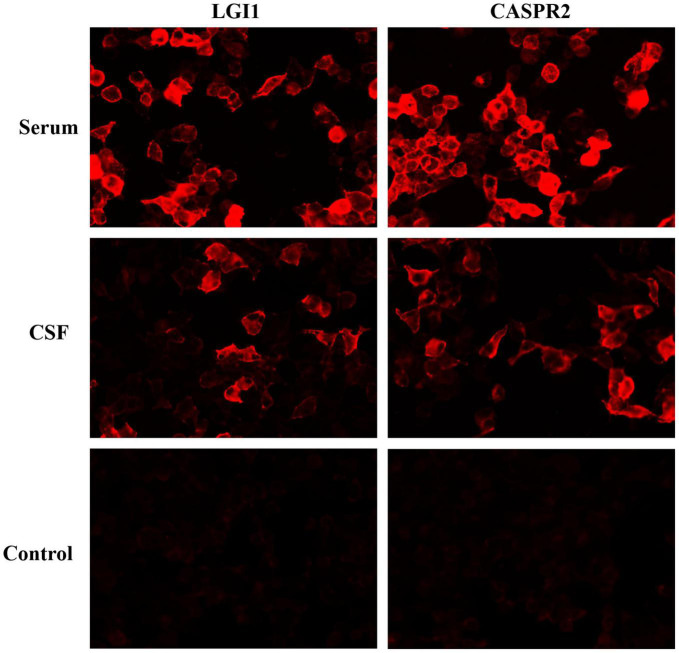
Positive reaction with transfected HEK293 cells expressing LGI1 and CASPR2 after incubation with serum and cerebrospinal fluid of patient 1 followed by anti-immunoglobulin secondary antibody.

Electroencephalography (EEG) showed slow-wave background, while magnetic resonance imaging (MRI) of the brain and spine were normal. No cardiovascular, renal, and endocrine system diseases causing arterial hypertension were found. Comprehensive screening for infectious and neoplastic disease on blood and CSF was unremarkable. CSF analysis was acellular with the average level of glucose and protein. Antineuronal antibodies screening revealed LGI1 antibodies (serum titer 1:30, CSF titer 1:10) and CASPR2 antibodies (serum titer 1:100, CSF titer 1:10), done by a commercial cell-based assay (CBA, Figure 1). The patient was diagnosed with Morvan syndrome, based on the clinical presentation of CNS (dyssomnia, irritability, and fatigue), PNS (pain), and autonomic nervous system (excessive sweating, tachycardia, hypertension) associated with serum and CSF LGI1 and CASPR2 antibodies.

Intravenous hydration and three antihypertensive drugs (captopril, sodium nitroprusside, metoprolol) were used as supportive treatment. Encephalopathy and dysautonomia had not been effectively improved. Immunotherapy was started immediately after diagnosis, 2 months after the onset of the disease. The patient received immunoglobulin intravenously (IVIG, 1 g/kg once daily for 2 days), methylprednisolone intravenously (IVMP, 20 mg/kg once daily for 5 days), and oral prednisolone (initial dose 1.5 mg/kg/day, slowly tapered over 3 months).

The patient started to improve gradually 1 week after immunotherapy. Both CNS and autonomic symptoms were significantly resolved at discharge 3 months after onset, except for mild hyperhidrosis and irritability resolved entirely about 1 month after discharge. The mRS score at discharge was 2 and was 0 2 months later. The patient had typical developmental milestones and no relapse 7 months after discharge.

#### Case 2

A previously healthy 3-year-8-month-old male presented with severe abdominal pain without vomiting and diarrhea for 2 weeks, followed by decreased appetite, fatigue, skin rash, pruritus, and excessive sweating. One month after the onset of abdominal pain, he developed behavioral change, manifested by shouting, episodes of crying, and irritability. The patient gradually showed sleep disorders, manifested by difficulty falling asleep and easy to wake up. He refused to walk because of lower limb pain. Within 2 months before admission, his weight lost 1.5 kg. Physical examination revealed skin rash, mainly in the lower limbs, tachycardia, arterial hypertension, and regular power in all limbs. The mRS score at admission was 4.

Blood and CSF samples screening showed no evidence for infectious and neoplastic disease. Neuroimaging (brain and spine MRI), computed tomography scans of the chest and abdomen, EEG, and electromyography were normal. There was acellular CSF with normal protein and glucose. Neural antibodies screening in serum detected positive LGI1 antibody (1:100) and CASPR2 antibody (1:100) by a commercial CBA. However, antibodies in CSF were negative. The diagnosis of Morvan syndrome was based on the combination of CNS (irritability, fatigue, episodes of crying, and dyssomnia), PNS (pain), and dysautonomia nervous system symptoms (tachycardia, skin rash, hypertension, and profuse sweating) as well as serum LGI1 and CASPR2 antibodies.

The case received symptomatic therapy with risperidone and two antihypertensives (captopril and nifedipine). However, the clinical manifestations were not improved. Immune therapy was started 2 months after the onset of symptoms. IVIG (1 g/kg once daily for 2 days) and IVMP (20 mg/kg/day for 5 days) followed by oral prednisone (initial dose 1.5 mg/kg/day) were administrated. Behavioral abnormalities and dysautonomia were slowly improved 2 weeks after immunotherapy. The mRS score 2 months after discharge was 0.

### Literature Review

#### Demographics and Clinical Symptoms

Fifteen patients were collected from seven publications (5, 6, 9, 12–15). Together with the 2 patients we presented, a total of 17 pediatric patients were identified, including 12 males and 5 females. At the onset, the median age was 4.1 years (range 1–16, interquartile range 2.5–13.5), with nine children younger than 5 years and six adolescents. The clinical characteristics are summarized in Tables 1, 2. Eleven patients were diagnosed with Morvan syndrome, four with acquired neuromyotonia, one with Guillain-Barré syndrome, and one with Guillain-Barré syndrome combined with Morvan syndrome. Dysautonomia (14/17, 82.3%), pain (13/17, 76.4%), sleep disorders (13/17, 76.4%), encephalopathy (12/17, 70.5%), and weight loss (10/17, 58.8%) were the most frequently described symptoms overall.

**TABLE 1:** Clinical characteristics of pediatric patients associated with double antibody-positive (LGI1 and CASPR2).

Patient, age/sex/(Ref)	LGI1/CASPR2 IgG serum	LGI1/CASPR2 IgG CSF	Phenotype	Clinical symptom				Comorbidities
				Encephalopathy	Dysautonomia	Neuromuscular symptoms/PNS	Other symptoms	
1, 4 y 1 mo/M/the present study	+/+	+/+	MoS	Irritability, fatigue	Tachycardia, hypertension, profuse sweating	Pain	Dyssomnia, weight loss	None
2, 3 y 8 mo/M/the present study	+/+	−/−	MoS	Irritability, fatigue, episodes of crying	Tachycardia, rash, hypertension, profuse sweating, pruritus	Pain	Dyssomnia, weight loss	None
3, 12 mo/F/(12)	+/+	+/+	MoS	Irritability, fatigue, decreased level of consciousness	Rash, hyperhidrosis, tachycardia, hypertension	Neuropathic itching	Insomnia, non-purulent conjunctivitis	None
4, 13 y/M/(9)	+/+	n.d.	aNMT	None	Hypertension	Pain, muscle cramps, neuromyotonia	Insomnia, weight loss	None
5, 14 y/M/(9)	+/+	n.d.	aNMT	None	None	Muscle cramps, neuromyotonia	Weight loss	Type 1 diabetes mellitus, membranous glomerulonephritis
6, 4 y/M/(9)	+/+	n.d.	aNMT	None	None	Pain, muscle weakness, neuromyotonia	Weight loss	MCAD deficiency, hyper-IgE syndrome
7, 13 y/F/(5)	+/+	n.a.	MoS	Seizures, behavioral changes, neuropsychiatric symptoms	Diaphoresis	Pain, muscle cramps	Insomnia, weight loss, abnormal gait	n.a.
8, 15 y/M/(5)	+/+	n.a.	aNMT	None	None	Pain, muscle cramps, fasciculations	None	n.a.
9, 6 y/F/(5)	+/+	n.a.	MoS	Behavioral changes, anxiety	Cardiac arrhythmia, hypertension	Pain	Insomnia, weight loss	n.a.
10, 16 y/M/(13)	+/+	−/+	MoS	Confusion, hallucination	Tachycardia, hypertension, profuse sweating, constipation	Pain, muscle weakness, tremor, muscle cramps	Insomnia, mild hyponatremia	None
11, 2 y/M/(14)	+/+	n.a.	GBS	None	Difficulty passing urine, constipation, skin flushing, excessive sweating, hypertension	Progressive weakness, abnormal sensation, hypotonia, facial weakness	None	n.a.
12, 3 y/F/(15)	+/+	−/−	GBS + MoS	Irritability, nocturnal restlessness, episodes of crying	Tachycardia, hypertension, hyperhidrosis, pruritus	Weakness	Sleep dysregulation, ataxia, mild hyponatremia	None
13, 1 y 6 mo/M/(6)	+/+	−/+	MoS	Behavioral change, agitated, episodes of unprovoked crying	Hypertension, pruritus, tachycardia, excessive sweating, constipation, pustulous palmar and plantar, erythema/eczema	Pain, suspected weakness of lower limbs, muscle hypotonia	Insomnia, sleep disturbance, weight loss, dysesthesia	n.a.
14, 1 y 8 mo/M/(6)	+/+	n.d./−	MoS	Reduced vigilance, behavioral change, unprovoked crying	Hypertension, pruritus	Pain, general weakness	Insomnia, weight loss, dysregulation of diurnal rhythm, appetite loss	None
15, 3 y/M/(6)	+/+	n.d.	MoS	Behavioral change	Hypertension, pruritus, tachycardia, excessive sweating	Pain, suspected general weakness	Sleep disturbance, weight stagnation	None
16, 6 y 4 mo/M/(6)	+/+	+/+	MoS	Reduced vigilance, behavioral change, restlessness, focal status epilepticus, drop attacks, agitation	Hypertension, pruritus, tachycardia	pain, weakness of the left arm	Insomnia, loss of vision	None
17, 15 y/F/(6)	+/+	+/+	MoS	Agitated, episodes of unprovoked crying, loss of orientation	Excessive sweating, pruritus, tachycardia, hypertension	Pain, myokymia	Insomnia, weight loss	None

*y, year; mo, month; M, male; F, female; n.d., not done; n.a., not available; MoS, Morvan syndrome; aNMT, acquired neuromyotonia; GBS, Guillain-Barré syndrome; MCAD, medium-chain acyl-CoA dehydrogenase; IgE, immunoglobulin E; PNS, peripheral nervous system.*

**TABLE 2:** Investigations and immunotherapy in pediatric patients associated with double antibody-positive (LGI1 and CASPR2).

Patient	CSF	MRI	EEG/ENG or EMG	Immune treatment	Supportive treatment	Treatment response	Follow-up (mo)	mRS score at acute phase/follow-up
1	Normal	Brain and spine normal	Slow wave/n.d.	IVIG, IVMP, Pred (3 mo)	Triple antihypertensive therapy	Complete remission	7	3/0
2	Normal	Brain and spine normal	Normal	IVIG, IVMP, Pred	Dual antihypertensive therapy	Complete remission	2	4/0
3	Normal	Brain and total body PET-MRI normal	Normal	IVMP, IVIG, Pred (5 mo)	Triple antihypertensive therapy, antihistaminic drugs	Complete remission	12	n.a.
4	Normal	Brain and spine normal	n.d./myokymia	None	Dual antihypertensive drugs, amitriptyline, paracetamol, codeine, gabapentin	Complete remission	1	n.a.
5	n.a.	Spine normal	n.a./normal	None	None	Complete remission	1	n.a.
6	n.a.	n.a.	n.a./myokymia	IVMP, IVIG, Pred, MTX	None	Partial remission, relapse, immunotherapy-dependent	36	n.a.
7	n.a.	Brain and spine normal	n.d./fasciculation potentials	IVIG	None	Partial remission	13	3/1
8	n.a.	Brain and spine normal	n.d./myokymia and fasciculation potentials	None	Symptomatic	Complete remission	15	1/0
9	Normal	Brain and spine normal	Normal	None	Symptomatic	Partial remission	6	3/1
10	High protein level^†^	Brain normal	n.a./demyelinating peripheral neuropathy, rare spontaneous discharges	IVMP, IVIG, Pred	Mirtazapine, pregabalin	Partial remission	1	4/1
11	High protein level^†^	Brain normal, spine: diffuse thickening and enhancement of ventral and dorsal roots within the cauda equina	n.a./features of a mixed motor and sensory demyelinating polyneuropathy	IVIG	None	Complete remission	3	n.a.
12	High protein level^†^	Brain normal, spine: diffuse thickening and enhancement of cauda equina nerve roots	Slow wave/n.a.	IVIG, IVMP, Pred	Triple antihypertensive therapy	Remission	1	n.a.
13	n.a.	Brain: diffuse T2 hyperintense white matter changes (6 and 8 w), normal body MRI	n.a.	IVIG, IVMP, Pred (4 mo)	Triple antihypertensive therapy, NSAIDs, PPI	Complete remission	8	n.a.
14	Mild pleocytosis	Brain and whole-body normal	n.a.	IVIG, IVMP, MMF (12 mo)	Quadruple antihypertensive therapy, NSAIDs, PPI, antihistamines	Remission, persisting ASD, delayed language development	17	n.a.
15	n.a.	Brain and spine normal	Disturbed sleep architecture/n.a.	IVIG, IVMP, Pred (3 mo)	Triple antihypertensive therapy, NSAIDs, PPI, antihistamines	Partial remission	3	n.a.
16	n.a.	Brain: bilateral posterior T2 hyperintensities with contrast enhancement (6 w)	Generalized slowing, ictal spike patterns and interictal occipital spikes/n.a.	IVMP	Quadruple antihypertensive therapy, antiepileptic therapy	Remission, mild attention deficits and mild behavioral change	16	n.a.
17	Mild pleocytosis	Brain and spine normal	Generalized slowing/n.a.	IVIG, IVMP, Pred (6 mo), RTX	Quadruple antihypertensive therapy, antihistamines, opioids, pregabalin	Complete remission	5	n.a.

*CSF, cerebrospinal fluid; MRI, magnetic resonance imaging; PET, positron emission tomography; EEG, electroencephalography; ENG, electroneurography; EMG, electromyography; mRS, modified Rankin Scale; mo, month; w, week; IVIG, intravenous immunoglobulins; IVMP, intravenous methylprednisolone; Pred, prednisone; MTX, methotrexate; MMF, mycophenolate mofetil; RTX, rituximab; n.a., not available; n.d., not done; NSAIDs, non-steroidal anti-inflammatory drugs; PPI, proton pump inhibitor; ASD, autism spectrum disorder.*

*^†^Cerebrospinal fluid analysis revealed high protein levels with normal cell count.*

Of the 12 patients with manifestations of Morvan syndrome (11 with Morvan syndrome and 1 with combined Morvan syndrome with Guillain-Barré syndrome), dysautonomia and sleep disorders occurred in all patients. Tachycardia, hypertension, and hyperhidrosis were the main manifestations of dysautonomia. Sleep disturbances and insomnia were common manifestations of sleep disorders. Encephalopathy occurred in 12 patients (12/12, 100%), primarily presenting behavioral changes (irritability, episodes of crying). Seizures only appeared in two patients (2/12, 16.6%). Pain, as the primary manifestation of peripheral nerve involvement, occurred in 10 patients (10/12, 83.3%), while 2 patients (2/12, 16.6%) also exhibited muscle cramps.

Four patients with acquired neuromyotonia were male and presented mainly with muscle cramps and neuromyotonia without CNS involvement, only one patient (patient 4) presented with sleep disturbances. One patient presented with classical Guillain-Barré syndrome with albuminocytologic dissociation and progressive limb weakness. One patient presented with Guillain-Barré syndrome combined with Morvan syndrome, with typical Guillain-Barré syndrome combined with encephalopathy and sleep disorders.

#### Investigations

All patients were positive for LGI1 and CASPR2 antibodies in serum. Eight patients were tested for both LGI1 and CASPR2 antibodies in CSF, and one patient only was tested for CASPR2 antibodies in CSF, four of whom had double-positive antibodies in CSF, two had CASPR2 antibodies only in CSF. A total of 13.3% (2/15) brain MRI was abnormal. Spinal MRI was abnormal in 14.2% (2/14) of patients. Five of the 10 patients with CSF testing were abnormal, including three with high protein levels and two with mild pleocytosis. No tumors were identified.

#### Treatment and Outcome

A total of 76.5% (13/17) of patients received immune therapy, including IVIG combination of IVMP during the acute symptomatic phase followed by oral prednisolone to maintain (*n* = 7), the combination of IVIG, IVMP, oral prednisolone, and methotrexate (*n* = 1), the combination of IVIG, IVMP, and mycophenolate mofetil (*n* = 1), the combination of IVIG, IVMP, oral prednisolone, and rituximab (*n* = 1), IVIG only (*n* = 2), IVMP only (*n* = 1). A total of 91.6% (11/12) of patients presenting with Morvan syndrome received immunotherapy. In contrast, 1 of 4 patients present with acquired neuromyotonia received immunotherapy. Four patients did not receive immunotherapy, three received supportive treatment, and one patient (patient 4) had spontaneous remission without any treatment. Median mRS scores in the acute phase were 3 (mean 3, range 1–4, data available in 6/17) and improved gradually. A total of 52.9% (9/17) patients recovered completely (median follow-up time 8.6 months, range 1–36 months). One patient (patient 6) relapsed over the follow-up period and was immunotherapy-dependent. No patient showed clinical signs of myasthenia gravis during disease and follow-up.

## Discussion

To date, no more than 70 cases of double-positive antibodies have been reported in adults and children (2, 6, 15, 16). In the study, we describe two pediatric Morvan syndrome with LGI1-CASPR2 double-positive and present the results of the first systematic literature review on pediatric LGI1-CASPR2 double-positive cases.

Previous studies have shown a significant male gender advantage in positive patients for either LGI1 or CASPR2 antibodies, or both antibodies (2, 12, 17). In our cases series, 12/17 (70.6%) were males, consistent with the condition reported in the literature review. In humans, CASPR2 mRNA was found at low levels in the ovary and prostate (18). Some patients suffered from CASPR2 antibodies disease after scrotal drainage (10). These studies suggest the possibility that the reproductive system may also contain these antigens in addition to brain tissue. However, the gender differences in the affected population remain unclear. Interestingly, although the age of onset in this group spanned infancy to adolescence, the vast majority of patients occurred before the age of 5 years (9/17, 52.9%) and during adolescents (6/17, 35.3%), which might represent an age-dependent feature of the syndrome.

Similar to adult patients, clinical presentations in our pediatric literature cohort with LGI1-CASPR2 double-positive presented different clinical syndromes with variable involvement of CNS, PNS, and autonomic nervous system (19). However, double-positive children had predominant peripheral and autonomic symptoms as previously described in the adult series (10, 19). In our pediatric literature cohort, the most frequent clinical syndromes included mixed central and peripheral symptoms, such as Morvan syndrome (11/17, 64.7%), and predominant PNS involvement, such as acquired neuromyotonia (4/17, 23.5%). Clinical syndromes with CNS involvement only, such as isolated epilepsy and limbic encephalitis, previously reported in adults, have not been described in pediatric patients (2).

The most common phenotypic spectrum of LGI1-CASPR2 double-positive associated neurological disorders in children is Morvan syndrome, similar to adults. Nineteen Morvan syndrome is recognized as a rare constellation of peripheral nerve hyperexcitability, dysautonomia, and encephalopathy with marked insomnia (10). Two patients in our center (case 1 and case 2) had various clinical syndromes involving CNS (encephalopathy and sleep disorders), PNS (pain), and autonomic 19 nervous system symptoms (hyperhidrosis, tachycardia, and hypertension) as well as serum and CSF LGI1 and CASPR2 antibodies. They were consistent with the clinical diagnosis of Morvan syndrome. In our pediatric literature cohort, 12 patients presented manifestations of Morvan syndrome (11 with Morvan syndrome and 1 with combined Morvan syndrome with Guillain-Barré syndrome), including dysfunction of the autonomic nervous system (12/12), sleep disorders (12/12), and encephalopathy (12/12, 100%) and pain (10/12, 83.3%). Muscle cramps were a common PNS symptom in adults with double-positive antibodies (2). Our literature review on Morvan syndrome with LGI1-CASPR2 double-positive disclosed only two pediatric cases (2/12, 16.6%) with muscle cramps, which the lower number of pediatric patients might partially explain. Seizures only appeared in two children (2/12, 16.6%). The incidence of epilepsy was lower in double-positive patients with CNS involvement (6, 10, 20, 21).

On the other hand, predominant PNS involvement is another typical clinical phenotype of double-positive children, similar to adults (21, 22). In our study, four children (4/17, 23.5%) with acquired neuromyotonia presented muscle cramps (3/4), neuromyotonia (3/4), pain (3/4), weight loss (3/4), muscle weakness (1/4), and fasciculations (1/4). One child presented with Guillain-Barré syndromes with typical features of albuminocytologic dissociation and progressive limb weakness. Patient 12 presented with Guillain-Barré syndrome combined with Morvan syndrome. Guillain-Barré syndrome has not been reported in adults with double-positive. However, CASPR2, as part of the VGKC-complex, is associated with neurological disease predominantly affecting the PNS (14, 23). In the previous report, Guillain-Barré syndrome was related to CASPR2 antibodies in children (14).

No underlying cancer was found in our double-positive children. Tumor occurs in up to 46% of adult patients with LGI1-CASPR2 double-positive (generally thymoma) in the previous reports (19). This is consistent with the fact that tumors in other neuroimmune diseases in children are lower than in adults. Two children had coexistent autoimmune disorders, one (patient 5) with type 1 diabetes mellitus and membranous glomerulonephritis, and another (patient 6) with medium-chain acyl-CoA dehydrogenase deficiency and hyper-IgE syndrome. In double-positive adult patients are prone to co-morbid myasthenia gravis (19).

Immunotherapy is currently widely used in antibody-mediated CNS disorders. For VGKC-related diseases, the limited available data are mainly retrospective and observational. Immunotherapies are the mainstay of treatment across all LGI1 and CASPR2 antibodies-associated syndromes. Immunotherapy is particularly effective than antiseizure medications in treating LGI1-antibodies-associated seizures (24). A total of 76.5% (13/17) of the patients received immunotherapy in this group. Glucocorticoids and IVIG were the initial regimens. Three were given second-line immunotherapy, including methotrexate, mycophenolate mofetil, or rituximab. Nine patients were treated with sequential oral prednisone acetate after IVMP. A total of 52.9% (9/17) achieved complete remission. The immunotherapy varied for different phenotypic spectrums. A total of 91.6% (11/12) of the patients with Morvan syndrome receiving immunotherapy were most often treated with glucocorticoids in combination with IVIG, 2 of whom were treated with IVIG or IVMP alone. A total of 41.6% (5/12) achieved complete remission. One patient with Guillain-Barré syndrome achieved complete remission with IVIG. Three of the four patients with acquired neuromyotonia were given non-immunotherapy to achieve complete remission, suggesting that immunotherapy is not mandatory for acquired neuromyotonia patients. Among the patients in partial remission, available information showed that three had a mRS score of 1 at the last follow-up. The description of sequelae symptoms was mainly cognitive or behavioral changes, which suggests a low probability of residual severe neurological deficits. Plasma exchange is effective immunomodulation in the acute phase of CNS autoimmune diseases, especially when corticosteroids are contraindicated or ineffective (25). However, limited evidence of efficacy and more adverse events has limited plasma exchange therapy in the pediatric population compared to steroids and IVIG (26).

## Conclusion

Our study delineates the clinical phenotype of pediatric patients with LGI1-CASPR2 double-positive, presenting multiple CNS, autonomic nervous system, and PNS symptoms. Morvan syndrome is the most common phenotypic spectrum. Although some patients do not achieve complete remission, immunotherapy can have a favorable prognosis. In acquired neuromyotonia, immunotherapy is not mandatory. Although the incidence of tumors in children is extremely low, screening is still necessary.

## Data Availability Statement

The original contributions presented in the study are included in the article/supplementary material, further inquiries can be directed to the corresponding author.

## Author Contributions

YJ, CT, TL, XS, JM, ZY, SH, XL, LJ, and YL contributed to the analysis and interpretation of data and references. YJ, CT, and YL participated in the conception and writing of the manuscript. All authors contributed to manuscript revision, read, and approved the submitted version

## Conflict of Interest

The authors declare that the research was conducted in the absence of any commercial or financial relationships that could be construed as a potential conflict of interest.

## Publisher’s Note

All claims expressed in this article are solely those of the authors and do not necessarily represent those of their affiliated organizations, or those of the publisher, the editors and the reviewers. Any product that may be evaluated in this article, or claim that may be made by its manufacturer, is not guaranteed or endorsed by the publisher.

## Footnotes

1
www.ncbi.nlm.nih.gov/pubmed
